# Omega-3 index as risk factor in psychiatric diseases: a narrative review

**DOI:** 10.3389/fpsyt.2023.1200403

**Published:** 2023-07-28

**Authors:** Helena Sofia Antao, Ema Sacadura-Leite, Narcisa Maria Bandarra, Maria Luisa Figueira

**Affiliations:** ^1^Faculty of Medicine, University of Lisbon, Lisbon, Portugal; ^2^CISP – Centro de Investigação em Saúde Pública, ENSP, Lisbon, Portugal; ^3^Centro Hospitalar Universitário de Lisboa Norte (CHULN), Lisbon, Portugal; ^4^Portuguese Institute for Sea and Atmosphere (IPMA), Lisbon, Portugal

**Keywords:** Omega-3 index, biomarker, risk factor, depression, bipolar disorder, psychosis, schizophrenia, dementia

## Abstract

Numerous studies have described associations between the omega-3 index (defined as the RBC percentage of EPA and DHA) and mental conditions, but no risk stratification or target value has gathered consensus so far. This narrative review aims to summarize the published data on the association between omega-3 index and mental illness and to contribute to the concept of an omega-3 index in the field of mental health. The bibliographic searches have been carried out in PubMed, Scopus and Web of Science databases to find relevant English language original research studies related to that association. The study search and selection process were registered in a PRISMA flow. Thirty-six studies were included in this review examining the links between omega-3 index and postpartum depression (3), major depression (15), major depression and bipolar disorder (1), bipolar disorder (4), schizophrenia and major depression (1), schizophrenia and other psychosis (5) and dementia (7). Thirty of these studies found either significant differences in omega-3 index between patients and controls or inverse relationships between omega-3 index and disease severity. The published evidence is compelling enough to suggest omega-3 index as a risk factor for some psychiatric diseases, specifically, major depression, postpartum depression, psychosis, and dementia. In occidental populations, we propose a risk threshold of (a) 4–5% in major depression and dementia, (b) 5% in postpartum depression, and (c) 4% for psychosis transition.

## 1. Introduction

The relationship of health with long-chain omega-3 fatty acids – also referred to as long chain polyunsaturated fatty acids or n-3 fatty acids (n-3 FA) - mainly eicosapentaenoic acid (EPA) and docosahexaenoic acid (DHA) – was discovered in 1979 (1) and a plethora of studies have been carried since then, reflecting an increasing interest in the prognostic value of their body levels (2).

The main dietary source of n-3 FA intake is fish (especially fatty species). While EPA and DHA can be synthesized from alpha-linolenic acid (ALA) in humans, most studies suggest that the conversion of ALA to EPA is less than 5% and the conversion to DHA is less than 0.05% (3). N-3 FA blood levels, metabolism and uptake from bloodstream can be affected by individual conditions and genetic determinants (4, 5).

Various means of measuring the expression of n-3 FA status have been used in research and clinical medicine. The omega-3 index (O3I) – defined as the sum of EPA and DHA content of red blood cells (RBC), expressed as a percentage of their total fatty acids - was initially proposed by Harris and von Schacky as a biomarker of the cardiac membrane content in omega-3 fatty acids and as a risk factor for coronary heart disease, especially sudden cardiac death. These authors concluded that the risk decreased by about 90% when O3I increased from 4% to values greater than 8% and defined a risk scale for death from coronary disease (0–4% high risk; 4–8%: intermediate risk; greater than 8%: desirable situation) (6).

O3I reflects other tissues’ content in EPA and DHA (7). It is acknowledged as a reliable long-term marker of n-3 FA intake and has shown to independently deliver predictive information for several illnesses (2). Depression, psychological stress, schizophrenia and dementia have been associated to the elevation of proinflammatory cytokines, including interleukin-1 beta, −2, −6 and − 18, interferon-gamma, and tumor necrosis factor alpha, which results in alterations of neurotransmitter precursors and metabolism as well as in activation of the hypothalamic–pituitary axis (8–10).

The role of n-3 FA in brain structure and functioning is not completely understood. Current knowledge has been largely built on the effects of their deficiency in animal models and on clinical trials that assessed the outcomes of supplementation in various psychiatric disorders. N-3 FA influence central nervous system health through molecular, cellular and neurobiological mechanisms (11) in three main areas – neuronal membranes, cytokine modulation and perfusion (12–15), all involved in the pathophysiology of depression, anxiety, schizophrenia and dementia.

As components of central nervous system membrane phospholipid acyl chains, n-3 FA are critical to their structure and function (16). Proteins embedded in the lipid bilayer of the cell act as transporters and receptors. Their conformation and, consequently, membrane fluidity, ion exchange, signaling and neurotransmission can be changed by n-3 FA (17, 18).

N-3 FA deficiency has been shown to alter the reservoir and release of serotonin and dopamine in animal models (19). The improvement in mood disorders associated with n-3 FA high levels may result from a modulation of membrane-bound receptors and enzymes involved in serotonergic neurotransmission and dopaminergic function (20) and better brain perfusion (21).

DHA has exhibited a protective effect against amyloid beta (Aβ) accumulation and its associated oxidative stress (15), inflammation, synaptic loss, tau protein hyperphosphorylation and the formation of neurofibrillary tangles (22) that occur in dementia.

The effects of n-3 FA supplementation on psychiatric illnesses have been studied in randomized clinical trials that are beyond the scope of this review. They have, nevertheless, brought complementary insights into the actions and mechanisms involved in n-3 FA interactions with the brain structure and function (23–26).

Despite numerous studies describing associations between O3I and mental conditions, no risk stratification or target O3I has so far gathered consensus in the field of mental health.

This narrative review aims to summarize the published data on the relationships between O3I and mental illness as well as to contribute to the concept of an O3I into the field of mental health raised by Milte et al. (27) and endorsed by other authors (28–32), helping physicians in identifying patients at higher risk and monitoring disease progression.

## 2. Materials and methods

The bibliographic searches were performed in PubMed, Scopus and Web of Science databases to find relevant English language original research studies evaluating associations between O3I and mental conditions. Each database was searched from inception to December 2022. These database searches validate exhaustive exploration and limit publication bias.

The primary search strategy was (“omega-3 index” OR “omega 3 index” OR EPA OR DHA) AND (depression OR anxiety OR bipolar OR schizophrenia OR psychosis OR attention OR hyperactivity OR dementia OR mental disease). One reviewer decided on the eligibility and data to extract, assisted in screening and selection by the web application Ryyan® 2022. Only publications including at least one association of O3I (described as such or as the percentage of EPA and DHA sum in total fatty acids) measured in RBC with a mental illness score calculated through a validated scale were included. Omega-3 supplementation clinical trials were included when baseline data was provided. The PRISMA flow diagram (33) was used to register the search steps and outcomes. The quality of the manuscript was evaluated by all the authors as per the SANRA six-item scale (34). As this review involved data from published studies only, institutional review board approval was not required.

## 3. Results

**Figure 1:**
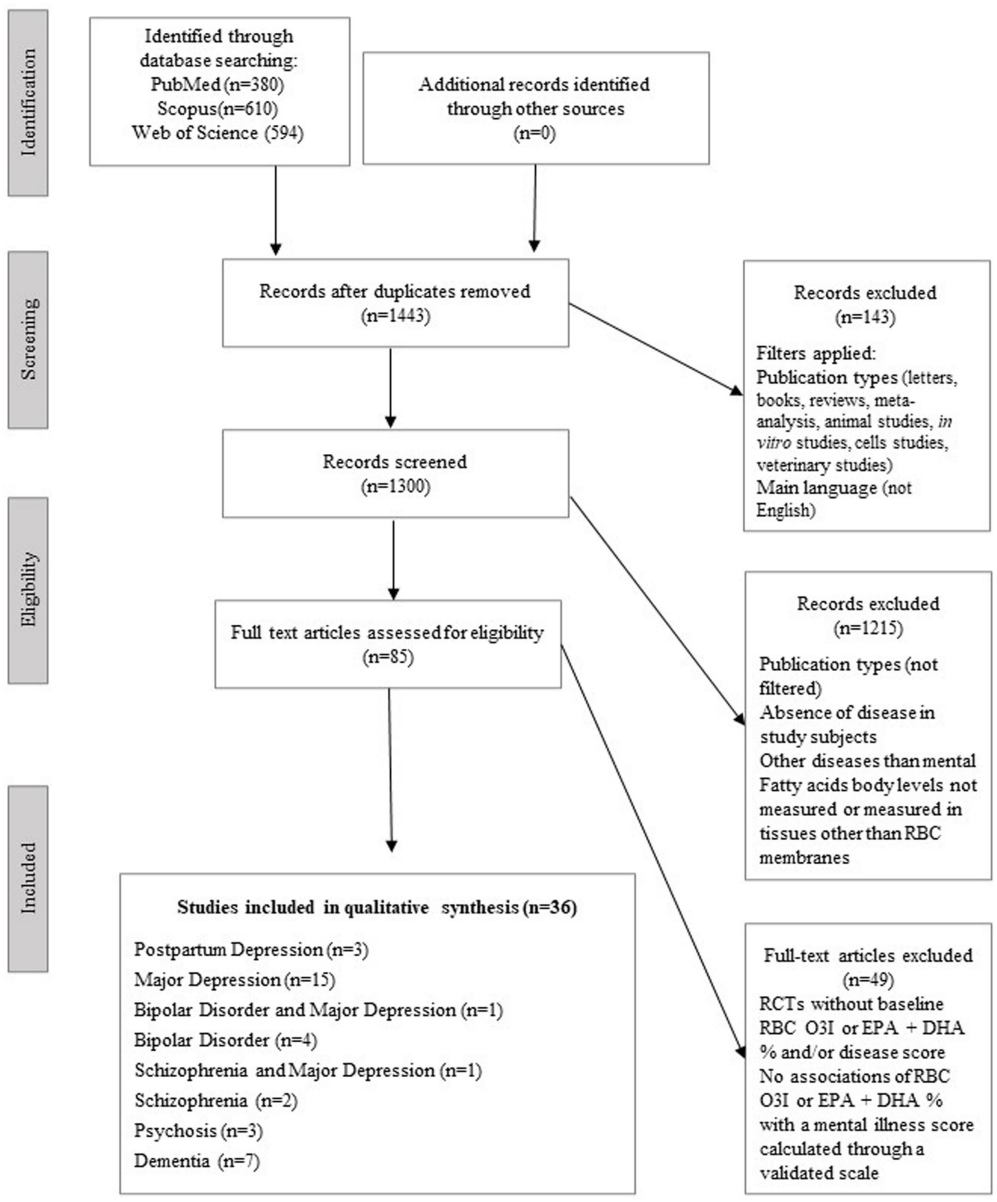
Study selection process (PRISMA flow diagram).

The study selection process is presented in Figure 1. Overall, thirty-six articles were included in the final qualitative analysis, published from 2008 to 2022. The design characteristics and major findings of each publication are presented in Table 1.

**Table 1:** Studies exploring relationships between omega-3 index and psychiatric diseases: designs and major findings.

Condition	Authors	Year	Study Design	Sample size	Country	Patients Population	Main O3I Outcome	Main Findings/ Conclusions
Postpartum depression	Markhus et al.	2013	cohort	35	Norway	*post partum* women	5% (cut-off)	the association between O3I and EPDS kinks at 5.1% (=QTR4) O3I was inversely associated with EPDS (*R*^2^ = 19)
Postpartum depression	Parker et al.	2015	cohort	821 87 cases +734 controls	Australia	*post partum* women	6.6% vs. 6.8% (mean)	O3I is only slightly linked with the risk of post-natal depression (quantified by the EPDS) no significant association was found in quantification by MINIAD
Postpartum depression	Hoge et al.	2019	cohort	71 17 cases +54 controls	Belgium	*post partum* women	5% (cut-off)	women with O3I < 5% had a 5-fold increased risk of depressive episode optimal O3I cut-off was 5.08%, with a sensitivity of 53% and a specificity of 83.3%
Major depression	Amin et al.	2008	case–control	759 118 cases +641 controls	U.S.A.	patients with confirmed ACS	4.8% vs. 5.5% (mean)	O3I was significantly reduced in depressed patients (4.8% vs. 5.5%) for each 4.54% rise in O3I, there was a 1-point decline in depressive symptoms (independent inverse association)
Major depression	Baghai et al.	2011	case–control	166 86 cases +80 controls	Germany	adult inpatients with MDD	3.9% vs. 5.1% (mean)	O3I was lower in MDD patients (3.9% vs. 5.1%) O3I was associated with high concentrations of IL-6
Major depression	Pottala et al.	2012	case–control	311 150 cases +161 controls	U.S.A.	adolescents admitted to hospital for depression	3.46% vs. 3.71% (mean)	inverse association between O3I and case status (unadjusted OR for case status was 0.72 for a 1% absolute increase in O3I)
Major depression	Park et al.	2012	cross sectional	168 80 cases +88 controls	Korea	depressed adults	8.61% vs. 9.47% (mean)	O3I was negatively associated with the risk of depression and correlated with CES-D score O3I could be a marker for depression in both low and high ranges.
Major depression	Sinn et al.	2012	RCT	50	Australia	people aged >65 years with MCI	r = 0·39	Improved GDS scores were correlated with increased O3I
Major depression	Baek et al.	2013	case–control	160 80 cases +80 controls	Korea	depressed adults	8.61% vs. 9.61% (mean)	O3I was lower in MDD patients; inverse association between CES-D-K and O3I O3I is negatively associated with levels of inflammation markers
Major depression	Johnston et al.	2013	cross sectional	78	U.S.A.	military service members with mild-to-moderate depression	3.45% (mean)	O3I was not associated with depression, anxiety or sleep scores O3I was directly associated with *CF* and EF especially in the 81% who reported poor sleep quality
Major depression	Grant et al.	2015	cross sectional	444	Australia	adolescents aged 16–18 years	5.9% (cut-off)	for participants with BMI > 23, having an OI < 5.9% (ideal 8%) increased the risk of depression no “severe to extremely severe” depression reported when O3I > 5.9% O3I was the 3^rd^ most significant predictor in the best classification model
Major depression	Schuchardt et al.	2015	RCT	111	Germany	50–80 yrs. old individuals with MCI	6.7% (cut-off)	lower omega-3 index was associated with depressive symptoms (BDI) In more than 85% of the patients, O3I was below 8% and in 23% below 5%
Major depression	Bigornia et al.	2016	cohort	787	U.S.A.	depressed adults with elevated oxidative stress biomarkers	β = −1.74 (top quartile)	inverse association between O3I and CES-D in participants with elevated oxidative stress biomarkers in the top tertile of urinary 8-OHdG, O3I was associated with significantly lower odds of a CES-D score ≥ 16
Major depression	Jin et al.	2016	cross sectional	214	Korea	postmenopausal women	9.94% vs. 10.47% (mean)	negative correlation between depression and O3I in women using HT no significant associations between erythrocyte FA and depression in women not using HT
Major depression	Cai et al.	2017	cross sectional	91	Australia	low fish consumers with heart disease and depressive symptoms	4.8% (mean)	no association between O3I and depressive symptoms no significant associations between O3I and angina status
Major depression	van der Wurff et al.	2018	cross sectional	257	Netherlands	LGSE adolescents aged 13–15 years	5% (cut-off)	no associations of O3I with depression or self-esteem scores in LGSE adolescents with O3I ≤ 5% the BF10 for O3I was <0.33, which indicates that there is more evidence for the H_0_, i.e., no association
Major depression	van der Burg et al.	2020	RCT	158	Australia	MDD adults with moderate-to-severe depressive symptoms	r = −0.458	change in EPA and DHA were significant predictors of change in MADRS O3I as a potential biomarker target to predict response to omega-3 treatment
Major depression	Cussotto et al.	2022	RCT	67	France, Germany, Spain	depressed adults	3.02% vs. 5.06% (mean)	lower O3I was associated to worse symptomatology responder to antidepressants had higher baseline O3I than non-responders
Bipolar disorder and Major depression	McNamara et al.	2010	cross sectional	60 20 MDD + 20 BD + 20 controls	U.S.A.	hospitalized patients with MDD and BD	3.8% (MDD) and 3.0% (BD) vs. 4.8% (mean)	MDD and BD patients had a lower O3I than controls 65% of MDD patients and 78% of BD patients had an O3I ≤ 4.0%, compared with 25% of controls
Bipolar disorder	Clayton et al.	2008	RCT	30 15 cases +15 controls	Australia	9–18 yrs. old adolescents with JBD	4.11% vs. 4.72% (mean)	O3I was not significantly different between groups
Bipolar disorder	Voggt et al.	2015	RCT	152 90 cases +62 controls	Germany	euthymic BD (I and II) patients	5.2% vs. 5.3% (mean)	O3I did not differ significantly between groups
Bipolar disorder	McNamara et al.	2016	cohort	130,102 cases +28 controls	U.S.A.	adolescents with BD I, MDD or family history (UHR)	4.0% (cut-off)	greater % of cases exhibit O3I ≤ 4.0% O3I is inversely correlated with manic and depressive symptom severity O3I may represent a promising prodromal risk biomarker
Bipolar disorder	Wulsin et al.	2018	cross sectional	172,116 cases +56 controls	U.S.A.	recent-onset BD I patients	3.4% vs. 3.9% (mean)	O3I was significantly lower in the bipolar group than in healthy subjects the rate of O3I deficiency (≤4%) was not significantly different between groups
Schizophrenia and Major depression	Parletta et al.	2016	pilot + RCT	130,116 MDD + 14 Schz	Australia	adults with schizophrenia or depression and CHD risk factors	3.95% (mean)	unfavorable O3I profile in people with schizophrenia or depression (average O3I = 3.95% vs. estimated 5% in the Australian population) no associations between O3I and mental health, quality of life or cardiometabolic health outcomes
Schizophrenia	McNamara et al.	2012	case–control	44 20 cases +24 controls	U.S.A.	adult medication free patients with schizophrenia	3.54% vs. 4.54% (mean)	patients O3I (3.5%) was significantly lower than healthy controls O3I (4.5%) the majority of SZ patients (72%) exhibited O3I ≤4.0% compared with 37% of controls
Schizophrenia	Li et al.	2022	cohort	486,327 cases +159 controls	China	15–60 yrs. olds with schizophrenia, schizoaffective or schizophreniform disorders	2.30% vs. 2.55% (mean)	baseline O3I was lower in patients than in controls O3I increased in the first episode group and decreased in relapsed patients O3I was a significant risk factor, correlated with treatment responsiveness
Psychosis	Alqarni et al.	2019	RCT	405,285 cases +120 controls	Australia, Austria, Denmark, Germany, Hong Kong, Netherlands, Singapore, Switzerland	young people at UHR for psychosis	2.99% vs. 4.09% (mean)	O3I is 26.9% lower in UHR O3I may be prodromal risk biomarker in the UHR population
Psychosis	Amminger et al.	2020	RCT	218	Australia, Austria, Denmark, Germany, Hong Kong, Netherlands, Singapore, Switzerland	young people at UHR for psychosis	2.77% vs. 3.17%s (mean, 6 M) 2.15% vs. 2.41% (mean, 12 M)	higher O3I baseline levels and increases predicted overall clinical improvement at 6 M and 12 M higher O3I baseline levels may exert longer-term protective effects
Psychosis	Allott et al.	2022	RCT	288	Australia, Austria, Denmark, Germany, Hong Kong, Netherlands, Singapore, Switzerland	young people at UHR for psychosis	VLM: 7.2% vs. 8.6% (mean) EF: 7.3% vs. 8.4% (mean)	higher baseline O3I was associated with a higher likelihood of membership in the extremely impaired class clinical variables including transition to psychosis were not associated with O3I
Dementia	Lukaschek et al.	2016	cross sectional	720	Germany	KORA-Age study participants aged 68–90 yrs	8.02% (target)	low O3I is associated with higher risk for mild cognitive impairment/suspected dementia (OR = 1.77) low O3I was a powerful predictor of cognitive impairment recommended O3I target: 8.02% ± 1.02%
Dementia	Ammann et al.	2017	cohort	6,706 587 PD + 571 MCI + 1,047 PD/MCI + 4,501 normal	U.S.A.	Women’s Health Initiative Memory Study participants	5.27% (mean) 1.52% (SD)	PD 15-yr cumulative incidence: 12.1% with high O3I (1 SD above mean) vs. 14.2% with low O3I (1 SD below mean) higher O3I may help protect against the development of dementia
Dementia	Coley et al.	2018	RCT	724	France, Monaco	MAPT participants	5.3% (cut-off)	lowest quartile of baseline O3I (≤4.83%) underwent significantly more 3-year cognitive decline than the other quartiles in a ROC curve analysis, the optimal omega-3 index cut-off for predicting notable cognitive decline was 5.3%
Dementia	Thomas et al.	2020	cohort	1,279	France	70 and older with a spontaneously reported memory complaint	4.6% (cut-off)	higher O3I was associated with lower risk of dementia, lower decline in cognition, memory and brain volume O3I <2.2% had a 1.6 times higher risk of dementia than O3I ≥4.6% an increase of 1.25% in O3I was associated with a 0.87 HR for dementia risk
Dementia	Rouch et al.	2022	cohort	832,272 MCI + 95 AD +465 normal	U.S.A.	Alzheimer’s Disease Neuroimaging Initiative participants	3.7% (cut-off)	longitudinally, low O3I was associated with greater Aβ accumulation and WMS cognitive decline but lower ADAS-Cog cognitive decline cross-sectionally, low O3I was associated with lower WMS cognition and higher tau accumulation among ApoE ε4 carriers
Dementia	Sala-Vila et al.	2022	cohort	1,490	U.S.A.	Framingham Offspring participants (dementia-free, ≥65 years old)	<4.3% vs. >7.1%	Q1 individuals (O3I < 4.3%) showed slightly attenuated risk to AD and all cause dementia progression than Q5 individuals (O3I > 7.1%) Using O3I (or RBC EPA) as the exposure of interest resulted in weaker and non-significant associations than DHA alone an increase in RBC DHA from Q1 to Q5 provides 4.7 additional years free of AD (higher benefit in APOE-ε4 carriers)
Dementia	Satizabal et al.	2022	cross sectional	2,183	U.S.A.	Framingham Offspring participants (dementia-free, ≥65 years old)	Q2-Q4 [3.9–5.3%] vs. Q1 (≤2.9%)	higher O3I (Q2-Q4 vs. Q1) was associated with larger hippocampal volumes (6.9 cm3 vs. 6.8 cm3) and better abstract reasoning (17.4 vs. 17.0 “similarities” scoring) higher levels of all omega-3 predictors were related to lower white matter hyperintensity burden but only in APOE-ε4 carriers

ACS, acute coronary syndrome; AD, Alzheimer disease; ADAS-Cog, Alzheimer disease assessment scale; cognitive subscale, BF -Bayes factor; BD, bipolar disorder; BMI, body mass index; CES-D(-K), center for epidemiologic studies depression scale (Korean population); CHD, coronary heart disease; EF, executive function; EPDS, Edinburgh postnatal depression scale; FA, fatty acids; GDS, geriatric depression scale; HT, hormonal therapy; IBOS, inmate behavior observation scale; iNOS, inducible nitric oxide synthase (iNOS); IL, interleukin; JBD, juvenile bipolar disorder; LGSE, lower general secondary education; MAPT, multidomain Alzheimer preventive trial; MCI, mild cognitive impairment; MDD, major depressive disorder; MINIAD, mini international neuropsychiatric interview and/or taking an antidepressant medication; O3I, omega-3 index; OR, odds ratio; RBC, red blood cells; PD, probable dementia; Q- quartile, RCT; randomized controlled trial, ROC; receiver operating characteristic, Schz – schizophrenia; TNF-α, tumor necrosis factor alpha; VLM, verbal and learning memory; WMS, Wechsler memory scale; β, effect size; r, Pearson correlation factor; ρ, Spearman correlation factor.

Thirty of these studies found either significant differences in omega-3 index between patients and controls or inverse relationships between omega-3 index and disease severity.

## 4. Discussion

The literature has yielded heterogeneous results with respect to the links between O3I and psychiatric disorders.

The articles returned by our search show a satisfactory diversity in populations’ age groups, baseline diseases and countries of origin. Most publications included in this review – albeit not all – showed a beneficial effect of higher O3I levels over disease severity in O3I lower ranges. Correlations between O3I and symptoms gravity were found in most studies where O3I was analyzed as a continuous variable. Those that compare O3I values of ill and healthy individuals, show small differences, usually not greater than 1%.

Although the diversity of methods, designs, sample sizes, O3I cut-offs and O3I target values proposed entangles the construction of an O3I as risk marker in mental health, relevant conclusions and suggestions for future research can be drawn.

In postpartum depression both Markhus et al. (29) and Hoge et al. (35) findings aid the concept of an O3I in mental health with a well-supported cut-off of 5% for low vs. high risk. Even though the variable antepartum depression has not been fully controlled by these authors [contrary to Parker et al. (36)], for the purpose of anticipating an increased risk of depression after delivery, an O3I < 5% in blood collected up to the 28th week of gestation should be considered a risk factor.

MDD is the psychiatric disease with the largest body of evidence involving inverse relationships with O3I analyzed both as a continuous and as a discrete variable (28, 37–47). Some of its evidence also add to the knowledge of the disease mechanisms, including the roles that inflammation (38, 41) and oxidative stress (44) play in its pathophysiology.

Authors whose work contradicts this inverse relationship (Cai et al. (48), van der Wurff et al. (49) and Johnston et al. (50)) explain their results on the grounds of methodologic imperfections, low depression severity in homogenous population or O3I values (high overall values and narrow ranges). Cai et al. (48) admit an O3I influence on depression below a threshold of 4–5%. In adolescent populations, van der Wurff et al. (49) propose that the deep changes in brain development, social and emotional behaviors could have offset the protective effect of n-3 FA. Their findings were not replicated by Pottala et al. (28) and Grant et al. (42) who underline their contributions to the expansion of an O3I concept to the field of psychiatry and highlight the role of O3I in depression predicting models.

Studies carried out in Korea (39, 41, 45) generally found significant O3I differences between ill and healthy individuals, with the exception of depressed menopausal women who were not under hormone therapy, explained by Jin et al. (45) as resulting from a previously described synergistic interaction between n-3 FA and estrogen. Despite the potential value of O3I as a marker of depression both in low and high ranges (39) we confine our discussion of an O3I in mental health to occidental populations, suggesting that more Asian populations should be included in future research.

Cussotto et al. (47) realized that O3I significantly predicted response to antidepressants. Considering Bigornia et al. (44), where associations with O3I have only been found within the top quartile of oxidative stress biomarkers concentration, the discussion of whether O3I usefulness as a risk marker is stronger in certain groups of patients ought to be continued.

Two out of five publications on bipolar disorder could not find a significant difference between groups [Clayton et al. (51) and Voggt et al. (52)]. Explanations provided include small sample size and difficulty in matching cases and controls. The authors also point out that both cases and controls had low O3I values, all in the range considered as medium risk for cardiovascular disease. McNamara et al. (53) suggest that an O3I ≤4% has the potential to become a prodromal risk biomarker and help to identify individuals at increased risk for bipolar disorder, underlining the low O3I levels found in these patients more as a risk indicator for sudden cardiac death than a mental disease marker.

The evidence provided by the remaining bipolar disorder studies (54, 55), although valuable and signaling a higher risk in individuals with lower O3I levels, is insufficient to propose O3I ≤4% as a prodromal risk of manic onset. The difference between percentages of patients and healthy controls with an O3I ≤4% has an overall weak clinical significance.

In the mixed schizophrenia and MDD population studied by Parletta et al. (56) (where schizophrenic patients were a minority) no associations between O3I and mental health outcomes were found. The authors compare the 3.95% O3I of their patients with the estimated 5% in the country’s population and underline the higher cardiovascular risk to which schizophrenia and MDD patients are exposed. Although they acknowledge the potential of O3I to estimate the risk of mental illness, their suggestions on future research are confined to the definition of supplementation targets and of critical periods for this intervention.

Despite the significant O3I difference shown by McNamara et al. (57) between schizophrenic patients and controls, the authors only discuss it as evidence of increased risk for cardiovascular morbidity and mortality.

Li et al. (58) findings fuel the debate about predicting antipsychotics responsiveness in schizophrenic patients raised by previously published papers that suggested biomarkers’ research could potentially eliminate resistance to antipsychotics (59).

The NEURAPRO trial (60) is the main available source of knowledge regarding relationships between O3I and the risk of psychosis onset in an ultra-high-risk population. Alqarni et al. (61) propose it as a prodromal risk biomarker. Due to the small effect size the concept of O3I < 4% as a risk factor for transition should be further discussed. Amminger et al. (62) suggest O3I as a prognosis marker, with higher O3I anticipating better clinical outcomes in the medium term but only discuss this finding in the context of n-3 FA supplementation and the effect size found is smaller (O3I approximately 0.5% higher in better responders). Finally, Allott et al. (63) could not find an association between O3I and clinical variables including transition to psychosis.

As to dementia, most of the longitudinal analyses showed a negative association between O3I and the probability of dementia and/or cognitive decline (32, 64–67). O3I effect size varied across studies as did cut-offs and target values proposed. Coley et al. (32) have suggested an O3I of 5% as protective against dementia (above which there would be no benefit), whereas Lukaschek et al. (64) recommended a target O3I of 8.02% ± 1.02%. Rouch et al. (67) have identified an O3I of 3.7% as a cut-off below which some dementia surrogate markers (e.g., higher Aβ and tau accumulation) were significantly increased. In analysis carried out within the Framingham offspring cohort, subjects aged 65 or over whose O3I was ≤2.9% had slightly smaller hippocampal volumes and showed worse abstract reasoning (68). Those whose O3I was <4.3% had a slightly lower risk to develop Alzheimer and all cause dementia, although DHA alone was a better predictor than O3I (69).

Based on the available literature, O3I seems to be a relevant and graded risk factor for the development of dementia with a stratification similar to proposed by Harris and von Shacky (6) for cardiovascular diseases.

This review has some limitations. Most of these studies were cross-sectional or based on cross-sectional analyses within studies with other designs. Causations cannot therefore be inferred.

Dichotomizations and/or risk stratifications could not be derived from several publications where O3I was analyzed as a continuous variable. On the other hand, O3I analyses as a discrete variable raise the questions of criteria for choices and loss of information. Examples of the latest are cut-offs previously adopted in non-psychiatric studies; it is not clear whether those are the most appropriate for the psychiatric arena. The proposal for an O3I in mental diseases should result from analyses carried out on raw data where different cut-offs could be tested.

In the light of most of the selected publications, O3I determination seems to be useful mainly to decide whether supplementation with n-3 FA is recommended. Its interest as an independent marker for diagnosis and independent predictor of response to medication and psychotherapy has not been, so far, exploited in depth.

## 5. Conclusion

Psychiatric disorders are typically heterogeneous. Several symptoms and phenotypes are shared across diagnoses and biological measurements do not separate completely from those of healthy individuals. This heterogeneity can be observed in the course of disease, in response to treatments and in genetic polymorphisms. Probably for these reasons, no psychiatric biomarker has been found so far (70).

It is still unclear whether O3I can be clinically useful as a marker for diagnosis and treatment response prediction. The published evidence is compelling enough to suggest omega-3 index as a risk factor for some psychiatric diseases, specifically, major depression, postpartum depression, psychosis, and dementia. In occidental populations, we propose a risk threshold of (a) 4–5% in major depression and dementia, (b) 5% in postpartum depression, and (c) 4% for psychosis transition (warranting further research). The concept of a protective O3I in mental health should be studied in more depth in forthcoming studies.

For the purpose of a more solid risk stratification, future research on these and other diseases should privilege analyses of O3I as a discrete variable and test several cut-offs.

## Author contributions

HA designed the study, defined the search flow, managed the literature searches, selected the articles, extracted and analysed the data with inputs from ES-L, NB, and MF. HA wrote the first draft of the manuscript. All authors contributed to the article and approved the submitted version.

## Conflict of interest

The authors declare that the research was conducted in the absence of any commercial or financial relationships that could be construed as a potential conflict of interest.

## Publisher’s note

All claims expressed in this article are solely those of the authors and do not necessarily represent those of their affiliated organizations, or those of the publisher, the editors and the reviewers. Any product that may be evaluated in this article, or claim that may be made by its manufacturer, is not guaranteed or endorsed by the publisher.
